# Cervical myelopathy due to neurovascular compression syndrome caused by persistent first intersegmental artery: a case report

**DOI:** 10.1186/s12883-020-01976-x

**Published:** 2020-11-03

**Authors:** Takuro Endo, Taku Sugawara, Naoki Higashiyama

**Affiliations:** Department of Spinal Surgery, Akita Cerebrospinal and Cardiovascular Center, 6-10 Senshu Kubotamachi, Akita-shi, Akita-ken, 010-0874 Japan

**Keywords:** Persistent first intersegmental artery, Neurovascular compression syndrome, Microvascular decompression

## Abstract

**Background:**

Persistent first intersegmental artery (PFIA) is a rare anatomical variation of vertebral arteries and is an asymptomatic finding in most cases. Here we report a rare case of cervical myelopathy caused by spinal cord compression by the PFIA.

**Case presentation:**

The patient was a 52-year-old man who complained of numbness and burning sensation around the neck and left shoulder area, partial weakness in the left deltoid muscle, right side thermal hypoalgesia, and disturbance of deep sensation since the past 1 year, and the symptoms had gradually worsened. Magnetic resonance imaging (MRI) and computed tomography (CT) showed spinal cord compression by the left PFIA at the C1/C2 level. Because conservative treatment was ineffective, microvascular decompression (MVD) of the PFIA was performed. The left PFIA was laterally transposed using polytetrafluoroethylene (PTFE) bands and anchored to the dura mater using three PTFE bands. To achieve adequate transposition, the small blood vessels bridging the spinal cord and PFIA and the dorsal root nerve had to be sacrificed. Postoperative T2-weighted MRI showed a small hyperintense region in the lateral funiculus of the spinal cord, but no new neurological deficits were identified. In the early postoperative stage, the patient’s deep sensory impairment and motor dysfunction were improved. His numbness and burning sensation almost disappeared, but slight thermal hypoalgesia remained in the lower limb.

**Conclusion:**

MVD is an effective treatment for spinal cord compression caused by the PFIA, but further studies are necessary to help address technical difficulties and avoid complications.

## Background

Persistent first intersegmental artery (PFIA) is an embryonic blood vessel that exists primarily during the early embryonic stage [1, 2]. However, in rare instances, PFIA persists in adults, with a frequency of approximately 0.7%, and is found as a unique blood vessel that enters the intradural space between the C1 and C2 laminae and runs a course along the spinal cord. This vertebral artery (VA) variation is diagnosed incidentally by angiography and is asymptomatic in most cases. Here we report a case of neurovascular compression syndrome (NVCS) caused by PFIA and treated using microvascular decompression (MVD).

## Case presentation

**Fig. 1:**
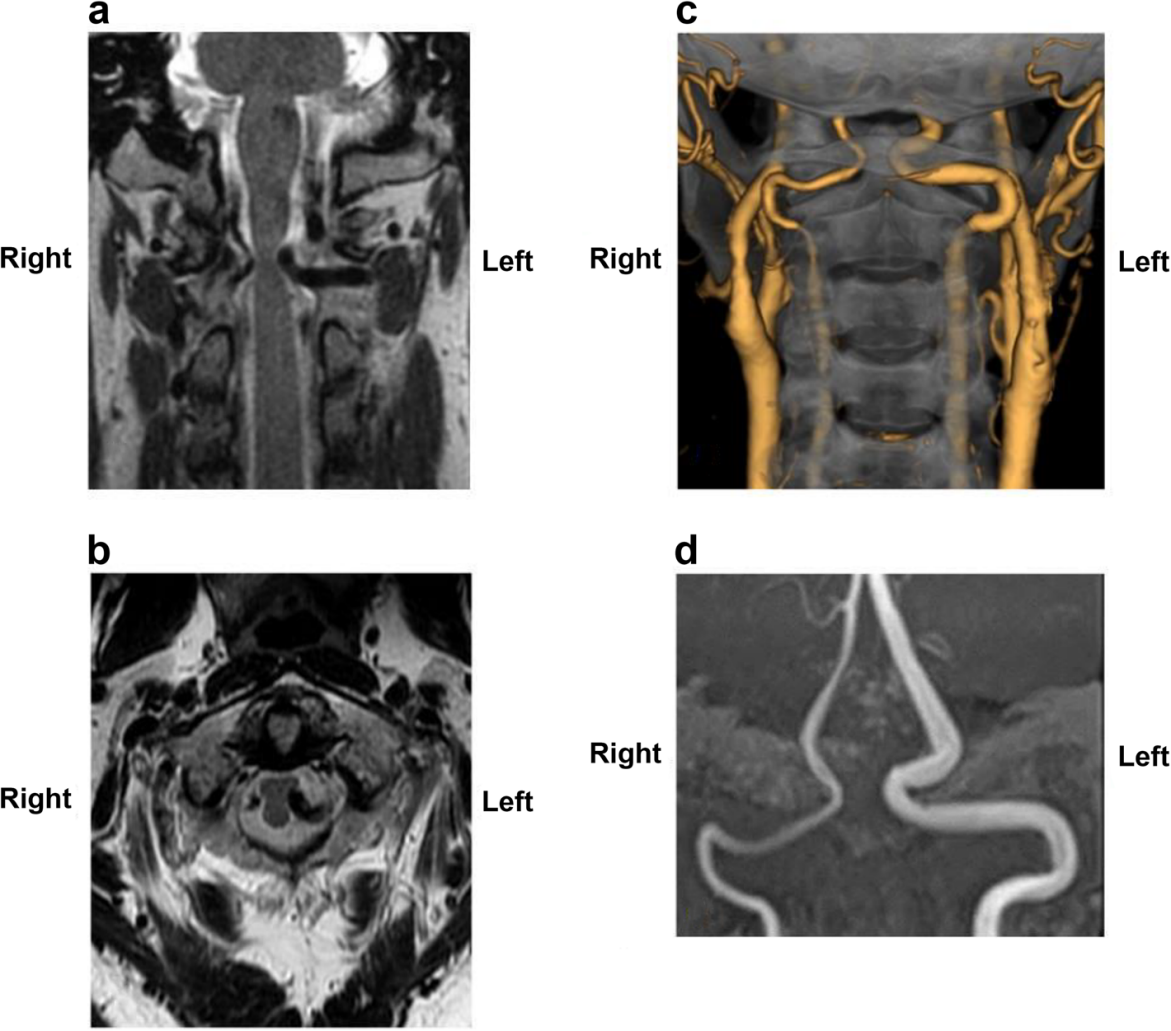
Preoperative radiological findings. Magnetic resonance image showing compression of the spinal cord caused by bilateral persistent first intersegmental artery (PFIA); the spinal cord was compressed strongly by the left PFIA (**a**, **b**). Both vertebral arteries pass through the foramen transversarium up to the C2 level and then branch as PFIAs on each side. Both PFIAs enter the spinal canal at the C1/C2 level and merge with each other at the distal side of the basilar artery (**c**, **d**). **a**: T2-weighted magnetic resonance image (coronal). **b**: T2-weighted magnetic resonance image (sagittal). **c**: Computed tomography angiography image. **d**: Magnetic resonance angiography image

The patient was a 52-year-old man with symptoms of numbness and burning sensation around the neck and left shoulder since the past 1 year. He had a history of hypertension and had already received adequate treatment with antihypertensive drugs before visiting our hospital. Neurological examination of the motor system revealed partial weakness in the left deltoid muscle, which was comparable to 4/5 on the Medical Research Council scale. Deep tendon reflexes in the biceps, brachioradialis, triceps, patellar, and Achilles tendons were normal. Neurological examination of the sensory system revealed thermal hypoalgesia of the right upper and lower limbs and body and disturbance of deep sensation, which manifested as a disability of toe and ankle position sensation and positive Romberg’s test. There was no obvious impairment of vibration sensation on both sides. Initial magnetic resonance imaging (MRI) showed spinal cord compression due to abnormal arteries that contacted the spinal cord bilaterally at the craniovertebral junction. Compression was particularly severe on the left side (Fig. 1a, b). Subsequent computed tomography (CT) angiography and magnetic resonance angiography revealed that the bilateral anomalous arteries passed through the foramen transversarium at the C2 level and entered the spinal canal between the C1 and C2 laminae. Finally, both abnormal arteries merged to form the basilar artery (Fig. 1c, d). The arteries corresponding to the normal VA did not exist; thus, these abnormal findings were diagnosed as PFIA. Disturbance of deep sensation and motor dysfunction remained unchanged; numbness, pain, and thermal hypoalgesia worsened; and conservative medical treatment with regular antihypertensive drugs, methylcobalamin, and nonsteroidal anti-inflammatory drugs was ineffective. Therefore, after a follow-up period of 20 months, surgical treatment was planned after obtaining the patient’s informed consent.

**Fig. 2:**
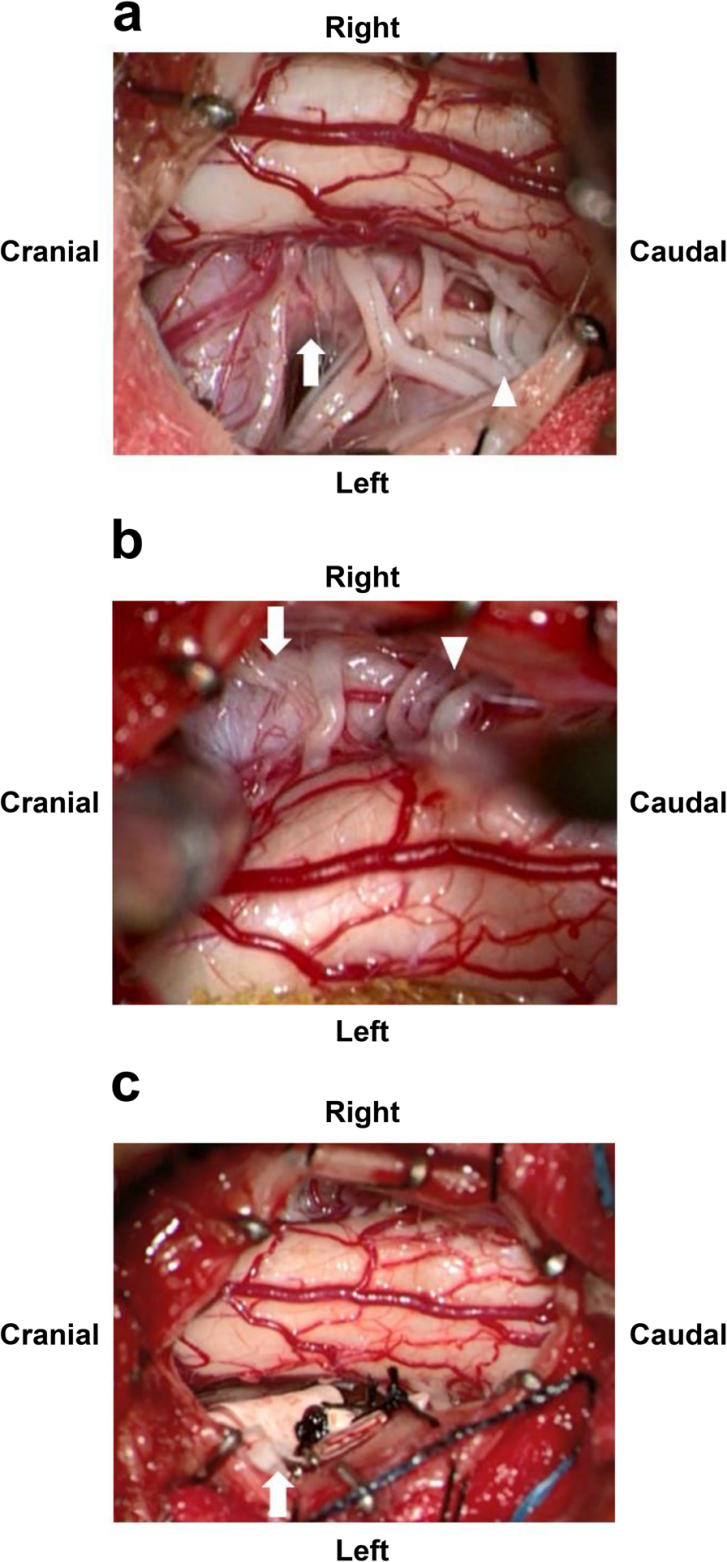
Intraoperative image. A linear skin incision was made from the inion to the C2 spinous process to expose the C1 posterior arch and C2 vertebral arch, and C1 laminectomy and C2 partial upper laminectomy were performed. The left persistent first intersegmental artery (PFIA) (white arrow) severely compressed the spinal cord at the C1 level. Left C2 dorsal root nerves (white arrow head) were observed around the left PFIA (**a**). The right PFIA (white arrow) course was also in contact with the spinal cord but with spinal cord compression. Right C2 dorsal root nerves (white arrow head) were observed around the right PFIA (**b**). The adhesion between the PFIA and surrounding vessels and nerves was dissected, and the PFIA was transposed using polytetrafluoroethylene bands (white arrow) and anchored to the dura mater. After transposition, sufficient space was established between the PFIA and spinal cord (**c**)

**Fig. 3:**
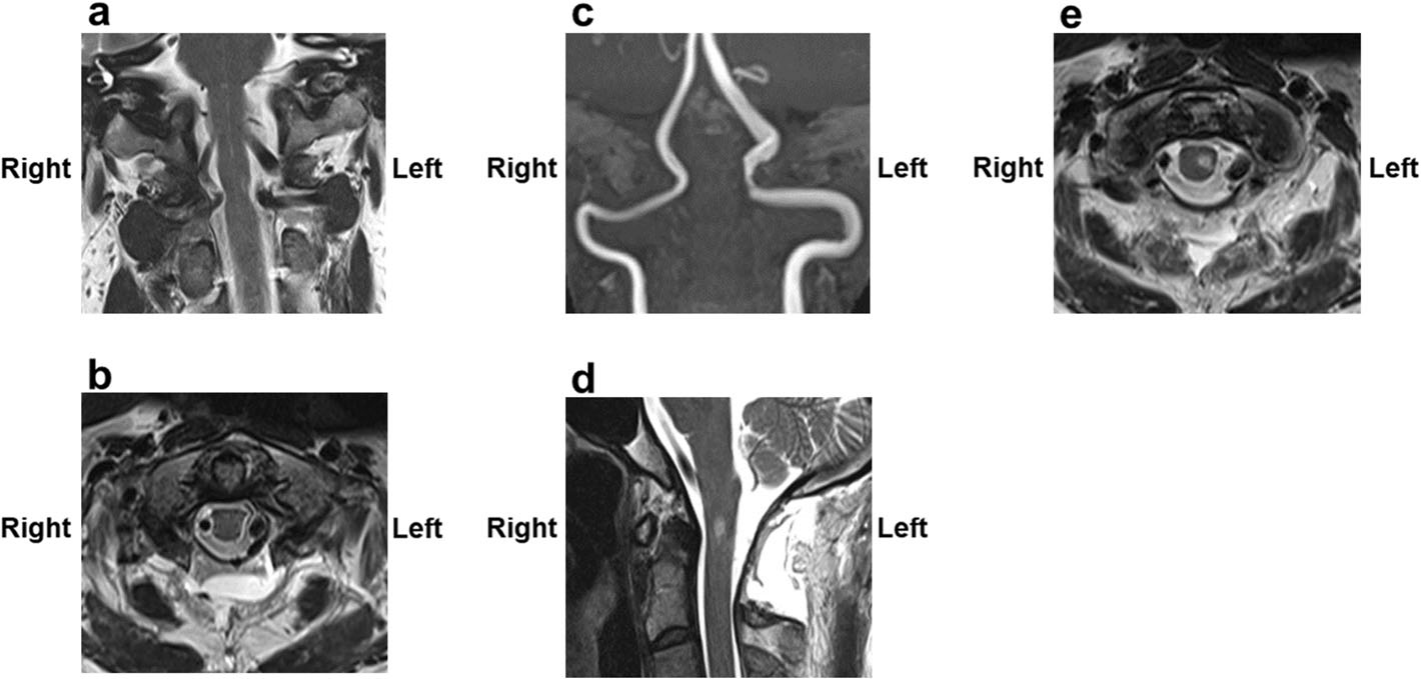
Postoperative radiological findings. The left persistent first intersegmental artery is moved away from the spinal cord, and compression of the spinal cord is reduced (**a**, **b**, **c**). A small hyperintense region is observed in the spinal cord on postoperative T2-weighted magnetic resonance images (**d**, **e**). **a**: T2-weighted magnetic resonance image (coronal). **b**: T2-weighted magnetic resonance image (sagittal). **c**: Magnetic resonance angiography image. **d**: T2-weighted magnetic resonance image (sagittal). **e**: T2-weighted magnetic resonance image (axial)

We performed MVD to translocate the left PFIA from the spinal cord. The patient was placed in the prone position, a 6-cm-long midline skin incision was made from the inion to the C2 spinous process, and a C1 laminectomy and C2 partial upper laminectomy were performed. As predicted by preoperative imaging, the left PFIA contacted and compressed the spinal cord to the extent that it formed an impression (Fig. 2a). The right PFIA also ran a course in contact with the spinal cord, but spinal cord compression was noted to be mild (Fig. 2b). We attempted to detach the adhesion between the spinal cord and left PFIA and separate the PFIA from the spinal cord by an interposition because medullary branches from the PFIA and dorsal root nerve were observed around the PFIA. However, the PFIA could not be separated from the spinal cord only by interposition, and transposition of the PFIA was necessary to sufficiently reduce spinal cord compression. Initially, we laterally pulled the left PFIA using a polytetrafluoroethylene (PTFE) band (Gore-Tex® sheet) with a width of 4 mm, and the PTFE band was then sutured to the dura mater using a 4–0 multifilament nonabsorbable polyamide suture. However, because of the stiffness and pulsation, PFIA was not effectively transposed. We subsequently added two additional PTFE bands to hold the PFIA in a wider area in the vertical direction, and the bands were anchored to the dura mater. To achieve adequate transposition, the small blood vessels bridging the spinal cord and PFIA as well as the dorsal root nerve were sacrificed. Finally, sufficient space was established between the PFIA and spinal cord (Fig. 2c). Transcranial electrical stimulation motor-evoked potential, free-run electromyograms, and triggered electromyograms were performed for intraoperative neurophysiological monitoring, and no abnormalities were identified before and after the sacrifice of the small vessels and nerves and transposition of the PFIA. Postoperative MRI showed that the PFIA was not in contact with the spinal cord and that spinal cord compression was resolved (Fig. 3a, b, c). Although a new, small hyperintense lesion was confirmed in the lateral funiculus of the spinal cord at the C1 level on T2-weighted MRI, no neurological deficits were observed (Fig. 3d, e). In the early postoperative stage, disturbance of deep sensation, which manifested as disability of toe and ankle position sensation and positive Romberg’s test, improved, and the patient could walk steadily. After 2 weeks of rehabilitation, the patient was able to walk with a normal gait without assistance. The numbness and burning sensation in his neck and left upper limb almost disappeared. Thermal hypoalgesia localized in the lower limb slightly persisted. Only slight weakness of the left deltoid persisted, but it was improved compared with the preoperative condition and compatible with normal function. The patient returned to work as a reinforcing bar placer 3 weeks after surgery, and there has been no apparent relapse of symptoms for 12 months after the procedure.

## Discussion and conclusions

PFIA is a VA variation with a unique course that penetrates the dura mater and enters the spinal canal at the C1/C2 level. Regarding the anatomical variations of VA at the craniocervical junction, Tokuda et al. reported that some variations were found in 2.3% of the studied sample [3]. Among these anatomical variations, the frequency of patients in whom the blood vessel corresponding to the normal running of VA regressed and only the first intersegmental artery remained was reported to be 0.67% [3]. The frequency of these unusual VA abnormalities increased to 19% if the patient showed bone malformations in the foramen of the skull and cervical vertebrae, which suggested that VA abnormalities are associated with sclerotome impairment [1]. Although a PFIA and the spinal cord are in contact along their course, the PFIA is only detected incidentally as an asymptomatic finding in most cases. This may occur because the subdural space is wider at the C1/C2 level than at other levels [4]. To the best of our knowledge, 15 cases of symptomatic PFIA have been reported to date [2, 5–18]. It is thought that the expansion and meandering of the blood vessel due to arteriosclerosis or pulsation of the artery due to hypertension are associated with the development of symptoms, so the symptoms often occur after middle age [5, 6]. Pain in the neck and upper limbs, accessory nerve paralysis, or cervical myelopathy has been reported as a clinical finding of NVCS associated with PFIA. Because of the apparent spinal cord compression by PFIA on images and corresponding neurological findings, we did not perform other examinations. However, neurophysiological examinations may help exclude other diseases, such as myopathy, neuropathy, inflammatory demyelinating and neurodegenerative disease of the spinal cord, and psychogenic disease [19]. In particular, symptomatic cases with spinal cord compression by PFIA are rare; therefore, careful preoperative evaluation is needed. The lack of objective information based on preoperative neurophysiological examination is a limitation in this report.

Few cases have been reported in which symptoms have been resolved with conservative treatment alone [18]. First, conservative treatment should be considered a priority, although there is no established medical treatment yet. As one of the mechanisms of NVCS, the water hammer effect by arterial pulsation has been suggested, and increased hemodynamic stress due to hypertension and hyperthyroidism has been reported to cause NVCS [5, 6, 18]. If the patient has such underlying diseases related to increased hemodynamic stress, control of disease condition by medications such as antihypertensive drugs may be considered, as well as analgesic or antiepileptic drugs. Transposition or interposition of the offending artery has been shown to be effective as a surgical treatment for NVCS, and these techniques are frequently used in surgery for hemifacial spasms or trigeminal neuralgia [20–22]. On the basis of findings in the literature and our clinical experience, MVD can be considered to be useful for NVCS in the spinal cord region [2, 5, 6, 10, 12, 14, 15].

In the present case, the PFIA was found to be bilateral, and strong spinal cord compression by the left PFIA was identified in the preoperative images. Intraoperatively, the medullary branches from the PFIA and dorsal root were intricately observed around the PFIA, so interposition was first considered. However, because the compression of PFIA against the spinal cord was quite strong, we assumed that the insertion of cushioning material would not provide sufficient decompression or further exacerbate compression by interposition; thus, we decided to perform transposition. For transposition of the PFIA, we used a method in which the offending artery was laterally moved and anchored to the dura mater using PTFE sheet material, as previously reported [5]. However, impaired blood flow in the medullary arteries due to kinking or extension of the PFIA by transposition has also been reported [6]. Small arteries feeding the spinal cord and branching from the PFIA had to be sacrificed to achieve sufficient decompression in our case. Following surgery, a small irregular hyperintense lesion appeared on T2-weighted MRI, probably caused by spinal cord ischemia due to sacrifice of the medullary branch. Fortunately, the lesion was asymptomatic, or, in case of any complications, only mild thermal hypoalgesia localized in the lower extremity was present; however, in other cases, such procedures may cause myelopathy. Especially in lesions such as those in this case, there was a possibility of occurrence of severe symptoms postoperatively; therefore, procedures that involve excessive and less prudent sacrifice of blood vessels must be strictly avoided. Accumulation of more surgical cases is necessary to optimize surgical procedures to prevent serious neurological complications and establish effective surgery.

In conclusion, we encountered a rare case of NVCS of the spinal cord involving a PFIA. Although there are only a few reports in the literature, MVD to alleviate spinal cord compression caused by PFIA was equally effective in this case as in the previously reported cases. However, a decision to sacrifice small arteries and dorsal nerve roots should be carefully considered, and further investigation and data accumulation are necessary to establish safer surgery.

## Data Availability

All data related to this case report are stored at Akita cerebrospinal and cardiovascular center (Akita, Japan) and are available from the corresponding author on reasonable request.

## Abbreviations

CT: Computed tomography
MRI: Magnetic resonance imaging
MVD: Microvascular decompression
NVCS: Neurovascular compression syndrome
PFIA: Persistent first intersegmental artery
PTFE: Polytetrafluoroethylene
VA: Vertebral artery
